# A Single δ^9^-Tetrahydrocannabinol (THC) Dose During Brain Development Affects Markers of Neurotrophy, Oxidative Stress, and Apoptosis

**DOI:** 10.3389/fphar.2019.01156

**Published:** 2019-10-04

**Authors:** Gaëtan Philippot, Erica Forsberg, Caroline Tahan, Henrik Viberg, Robert Fredriksson

**Affiliations:** ^1^Department of Organismal Biology, Environmental Toxicology, Uppsala University, Uppsala, Sweden; ^2^Department of Pharmaceutical Biosciences, Molecular Neuropharmacology, Uppsala University, Uppsala, Sweden

**Keywords:** δ^9^-tetrahydrocannabinol, developmental toxicology and neurotoxicology, acetaminophen (paracetamol), *Trkb*, oxidative stress, brain growth spurt, critical window development, neurotrophic

## Abstract

δ^9^-tetrahydrocannabinol (THC) is one of the most used drugs during pregnancy and lactation and efficiently crosses the placental and blood–brain barriers. Despite the recent legalization initiatives worldwide, the adverse outcome pathway (AOP) of THC following exposure during brain development is incompletely understood. We have previously reported that a single injection of THC on postnatal day (PND) 10 altered adult spontaneous behavior and habituation rates in adult mice. Similar behavioral alterations have been reported following PND 10 exposure to the commonly used over-the-counter analgesic acetaminophen (AAP; also known as paracetamol); as both THC and AAP interact with the endocannabinoid system, we hypothesize that this system might be involved in the AOP of both these pharmaceuticals/drugs. Here, we report that a single THC dose on PND 10 decreased transcript levels of *tropomyosin receptor kinase b* (*Trkb*) 24 h after exposure in both the frontal and parietal cortex, and in the hippocampus in mice. An increase in the *nuclear factor* (*erythroid-derived 2*)-*like 2* (*Nrf2*)/*Kelch-like ECH-associated protein 1* (*Keap1*) ratio were also found in both the parietal cortex and hippocampus following neonatal exposure to THC. In addition, THC exposure increased transcript levels of *cannabinoid receptor type 1* (*Cb1r*) in the parietal cortex and increased the apoptosis regulator BAX in the frontal cortex. This study is important for mainly 3 reasons: 1) we are starting to get information on the developmental neurotoxic AOP of PND 10 exposure to THC, where we suggest that transcriptional changes of the neurotrophic receptor *Trkb* are central, 2) our PND 10 exposure model provides information relevant to human exposure and 3) since PND 10 exposure to AAP also decreased *Trkb* transcript levels, we suggest THC and AAP may share key events in their respective AOP through endocannabinoid-mediated alterations of the brain-derived neurotrophic factor (BDNF)-TRKB signaling pathway.

## Introduction

There is an urgent need for scientific data regarding both the harm and benefit of cannabis use, because the use of this drug — both therapeutically and recreationally — is growing dramatically. There is already a large body of evidence concerning the potential cognitive and affective side-effects associated with cannabis exposure during brain development. In animal studies, pre- and perinatal cannabinoid exposure have been shown to affect locomotor activity, memory, social interaction, and emotional functions (Campolongo et al., 2011; Higuera-Matas et al., 2015; Vargish et al., 2016). Similar adverse effects have been observed in humans following developmental exposure to cannabinoids: altered cognition and executive functions, higher levels of depression and anxiety during adolescence (Higuera-Matas et al., 2015; Grant et al., 2018). In mammals, there is a period of time during brain development with increased vulnerability to toxic insults called the brain growth spurt (BGS) (Davison and Dobbing, 1968). In humans, the BGS starts during the beginning of the third trimester of pregnancy, continuing through its peak around birth and lasts until the 2–3 years of age (Davison and Dobbing, 1968; Dobbing and Sands, 1979). However, this period of time is postnatal in mice and starts a few days after birth and continues 2–3 weeks, with a peak around postnatal day (PND) 10 (Davison and Dobbing, 1968). During the peak of the BGS, many fundamental processes occur in the brain: dendritic and axonal outgrowth, synaptogenesis, establishment of neuronal connections, proliferation of glia cells, and myelination (Davison and Dobbing, 1968; Kolb and Whishaw, 1989). In mice, it has been demonstrated in numerous studies that single-day exposures to xenobiotics during the peak of the BGS can have long lasting effects on memory, learning, and locomotor activity (Eriksson, 1997; Viberg et al., 2008; Pontén et al., 2011; Viberg et al., 2013; Viberg et al., 2014).

The major psychoactive ingredient in cannabis is δ^9^-tetrahydrocannabinol (THC), which efficiently crosses the placental and blood–brain barriers, and — following consumption — can be found in milk of breastfeeding mothers (Schou et al., 1977; Perez-Reyes and Wall, 1982; Hutchings et al., 1989). The main target of THC is the cannabinoid receptor type 1 (CB_1_R); this receptor is already present during early brain development and is involved in both progenitor cell proliferation and differentiation, neural migration, synaptogenesis, and correct neurite and axonal outgrowth (Berghuis et al., 2005; Berghuis et al., 2007; Harkany et al., 2007; Harkany et al., 2008; Mulder et al., 2008). A previous study demonstrated that a single dose of THC (10 and 50 mg/kg) injected into 10-day-old mice was enough to alter their adult spontaneous behavior and change their habituation rates when introduced to a new home cage (Philippot et al., 2016). However, the neurotoxic mechanisms that are responsible for these PND 10 induced behavioral effects are still unknown. Interestingly, PND 10 exposure to the commonly used over-the-counter analgesic acetaminophen (AAP; also known as paracetamol) has also been proven to affect adult spontaneous behavior and habituation rates when introduced to a novel home cage (Viberg et al., 2014; Philippot et al., 2017). This is interesting because AAP, like THC, also interacts with the endocannabinoid system (Bertolini et al., 2006). Moreover, the neurotoxic effects following PND 10 AAP exposure were enhanced following co-exposure to CB_1_R agonist WIN 55 212-2 (WIN) (Philippot et al., 2018).

The legalization initiatives, and the fact that the endocannabinoid system is an emerging target of pharmacotherapy, increase the need for a thorough risk–benefit assessment of THC, where scientific evidence is crucial. Most existing experimental data on the developmental effects of cannabinoids originates from studies in rodents exposed to cannabinoids for several days or weeks. This is problematic because — in terms of key neurodevelopmental processes — weeks of exposure in rats and mice correspond to months of continuous exposure in humans, making these experimental conditions unrealistic and not representative to most exposure situations in humans. Primarily, this study aimed to investigate the possible key events in the adverse outcome pathway (AOP) in relation to our previous study (Philippot et al., 2016) where we showed that a single-dose of THC administered to 10-day-old mice induced long-term changes in their behavior. Secondly, the aim of this study was to compare these potential biochemical effects to those already observed following PND 10 AAP exposure (Philippot et al., 2018). Accordingly, a thorough analysis of the transcriptional changes following PND 10 exposure to THC was made in mice. More specifically, this study investigates transcript levels of genes associated with neurotrophic, endocannabinoid and oxidative stress signaling, together with genes associated with synaptic density, in the frontal and parietal cortex and in the hippocampus. In addition, this study investigated if exposure induced apoptosis in these brain regions. Our exposure model therefore provides toxicological data relevant to humans and that are highly warranted during times when exposure to cannabinoid receptor agonist is likely to increase.

## Materials and Methods

Experiments were conducted in accordance with the Directive of European Parliament and of the Council of 22 September 2010 (2010/63/EU), after approval from the local ethical committee (Uppsala University and Agricultural Research Council).

### Animals, Chemicals, and Exposures

Pregnant NMRI mice (from Charles River Laboratory) were purchased from Scanbur, Sollentuna, Sweden and were housed individually in plastic cages in a temperature-controlled (22°C) and light-controlled (12 h light/dark cycle) room with a relative humidity in the range 45–65%. All experimental animals had free access to standardized pellet food (Lactamin, Stockholm, Sweden) and tap water. The pregnant NMRI mice were checked for birth once daily (18.00 h) and day of birth was counted as PND 0. Within 48 h after birth, litter sizes were adjusted to 10–12 pups of both sexes.

THC (Dronabinol, LGC, DAC Quality) was generously provided by Pronexa and was dissolved in peanut oil/egg lecithin (Merck, Darmstadt, Germany). This mixture was sonicated with water to yield a 20% (w/w) fat emulsion. Stock solutions containing 2 and 10 mg THC/ml were made. Mice were subcutaneously injected once on PND 10 with either 10 or 50 mg THC/kg, or vehicle only, in the scruff. The doses of 50 and 10 mg THC/kg were chosen since these doses gave spontaneous behaviour alterations and changes in habituation capabilities (when introduced to a novel home cage) later in life, as previously reported (Philippot et al., 2016). Doses between 5–10 mg/kg are estimated to correspond to a moderate dose of THC and therefore have been used to mimic human exposures to THC (Campolongo et al., 2011).

Male pups, randomly selected from different litters, were administered subcutaneously with either 10 or 50 mg THC/kg or vehicle on PND 10. Pups were euthanized by decapitation 24 h after exposure and brains were dissected on an ice-cold glass plate where frontal cortex, parietal cortex and hippocampus were collected and individually snap frozen in liquid nitrogen and then stored at −80°C until assayed.

### Quantitative Real-Time Polymerase Chain Reaction (qPCR)

Relative expression levels of mRNAs were measured by quantitative real-time PCR. Brain regions were homogenized in PureZOL isolation agent (BioRad, Stockholm, Sweden). The total RNA was isolated using Aurum Total RNA extraction columns (Bio-Rad, Stockholm, Sweden) and treated with DNAse to remove possible genomic DNA contamination, according to the manufacturer’s instructions and stored at −80°C. The isolated RNA was checked for its purity and quantity using a Nanodrop 2000 UV-light spectrophotometer (Thermo fisher Scientific, Göteborg, Sweden) and RNA quality was checked (after denaturation with formamide) with agarose gel electrophoresis (1% agarose gel).

Three microgram of RNA was reverse transcribed to cDNA using iSCRIPT (BioRad) in 60 µl sample sizes and stored at −20°C. Measurement of gene expression was performed using quantitative real-time PCR on a C1000 Thermal Cycler with iQ SyBR Green Supermix (Bio-Rad, Stockholm, Sweden). Gene transcription of *brain-derived neurotrophic factor* (*Bdnf*), *tropomyosin receptor kinase B* [*Trkb*; encoded by *neurotrophic receptor tyrosine kinase 2* (*Ntrk2*)], *synaptophysin* (*Syp*), *postsynaptic density protein 95* [*Psd-95*; encoded by *large homolog 4* (*Drosophila*) (*Dlg4*)], *cannabinoid receptor type 1* [*Cb1r*; encoded by *cannabinoid receptor 1* (*Cnr1*)], *fatty acid amide hydroxylase* (*Faah*), *nuclear factor (erythroid-derived 2)-like 2* (*Nrf2*; encoded by *Nfe2l2* gene), and *Kelch-like ECH-associated protein 1* (*Keap1*) was normalized against transcription of housekeeping genes *phosphoglycerate kinase* 1 (*Pkg-1*) and *histone cluster 1 H3a* (*H3a)* for each sample. The gene specific PCR-primers are listed in **Table 1**. Each sample was run as a triplicate. The design of the reaction was 30 s denaturation at temperature 95°C, additional 55 repeats of denaturation for 10 s at 95°C, 30 s annealing at optimized temperature for every primer pair (**Table 1**) and elongation for 30 s at 72°C. To ensure the amplification of a single product, a melt curve for each qPCR reaction was performed (melt curve ranging from 55–95°C). Gene transcription analyses of the genes of interest were analyzed with the 2^−ΔΔCt^ method (Livak and Schmittgen, 2001).

**Table 1:** Gene-specific primer sequences used for qPCR.

Gene name	Forward primer (5′-3′)	Reverse primer (5′-3′)	Annealing temperature (°C)
*Bdnf*	GAAGAGCTGCTGGATGAGGAC	TTCAGTTGGCCTTTGGATACC	67.5
*Trkb*	TGGACCACGCCAACTGACATT	GAATGTCTCGCCAACTTGAG	70.0
*Psd95*	TCTGTGCGAGAGGTAGCAGA	AAGCACTCCGTGAACTCCTG	64.5
*Syp*	ACAGCAGTGTTCGCTTTCA	GGGTCCCTCAGTTCCTTG	56.0
*Cb1r*	TGAAGTCGATCTTAGACGGCC	GTGGTGATGGTACGGAAGGTA	60.9
*Faah*	GCCACACGCTGGTCCCCTTC	AGAGCAGCCACCATCACTGAACAG	64.5
*Nrf2*	GCCCACATTCCCAAACAAGAT	CCAGAGAGCTATTGAGGGACTG	64.5
*Keap1*	TGCCCCTGTGGTCAAAGTG	GGTTCGGTTACCGTCCTGC	67.5
*H3a*	CCTTGTGGGTCTGTTTGA	CAGTTGGATGTCCTTGGG	58.0
*Pgk1*	CTCCGCTTTCATGTAGAGGAAG	GACATCTCCTAGTTTGGACAGTG	58.0

### ELISA

The different brain regions were homogenized in 1 ml PBS (with phosphatase/protease inhibitor)/100 mg tissue with a bullet blender. Homogenates were centrifuged for 7 min at 5,000 rpm at 4°C. The supernatants were collected and stored at −20°C until use. Total protein concentrations were measured using the Protein Quantification Kit-Rapid (Sigma-Aldrich, USA). Apoptosis following 10 mg THC/kg [LOAEL in previous study (Philippot et al., 2016)] was assessed by measuring the levels of bcl-2-like protein 4 (BAX) levels in the frontal cortex, the parietal cortex and in the hippocampus 24 h after exposure using Mouse Bax ELISA Kit ELISA kits (ab233624) according to the manufacturer’s instructions (Abcam; Cambridge, UK).

### Statistical Analysis

Normality of residuals and homogeneity of variance were tested for using QQ-plots and homoscedasticity-plots (accompanied by Bartlett’s test), respectively; if needed, data were log-transformed to meet the assumptions of parametric statistics. If assumptions as such were fulfilled, one-way ANOVAs followed by Tukey’s multiple comparisons test or Student’s t-test were used. If not, Kruskal-Wallis test followed Dunn’s multiple comparisons test were used. More detailed information on what test that was used is provided in results section and figure legends. Estimation of effect sizes was done using partial eta-squared (η_p_^2^) and Cohen’s d (*d*). Graphical illustrations, normality testing and analyses of equal variances were made in GraphPad Prism version 8.0.2 (GraphPad software Inc., CA, USA).

## Results

### qPCR

**Figure 1:**
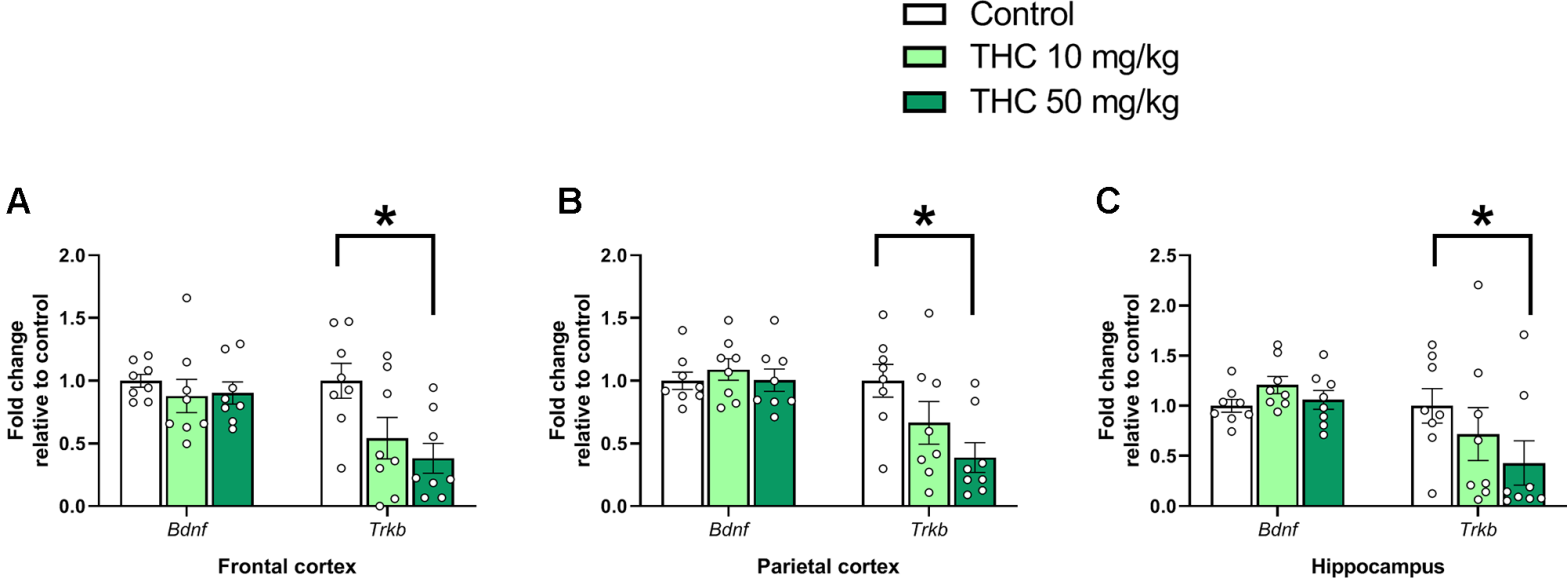
Neurotrophic gene markers. Transcript levels of neurotrophic genes *Bdnf* and *Trkb* 24 h after PND 10 exposure to vehicle, 10 mg THC/kg or 50 mg THC/kg in the **(A)** frontal cortex, **(B)** parietal cortex, and **(C)** hippocampus. Statistical difference from *post hoc* test is indicated as * if p < 0.05. Height of bars represents the mean fold change ± SEM of 8 mice.

Analysis of variance demonstrated an effect of THC exposure on relative expressions of *Trkb* in both frontal cortex [F (2, 21) = 5.08, *p* = 0.016, η_p_^2^ = 0.33] and parietal cortex [F (2, 21) = 4.72, *p* = 0.0203, η_p_^2^ = 0.31]. *Post hoc* analyses using Tukey’s multiple comparisons test revealed a decrease in *Trkb* gene transcript levels in mice neonatally exposed to 50 mg/kg compared to controls in both frontal cortex (*p* = 0.015) and parietal cortex (*p* = 0.016) (**Figures 1A, B**). Tukey’s multiple comparison test also revealed a significant decrease of *Trkb* gene transcription in the hippocampus in mice exposed to 50 mg/kg compared to controls (*p* = 0.041); however, no main effect was observed in the variance analysis [F (2, 21) = 3.44, *p* = 0.051, η_p_^2^ = 0.25] (**Figure 1C**).

**Figure 2:**
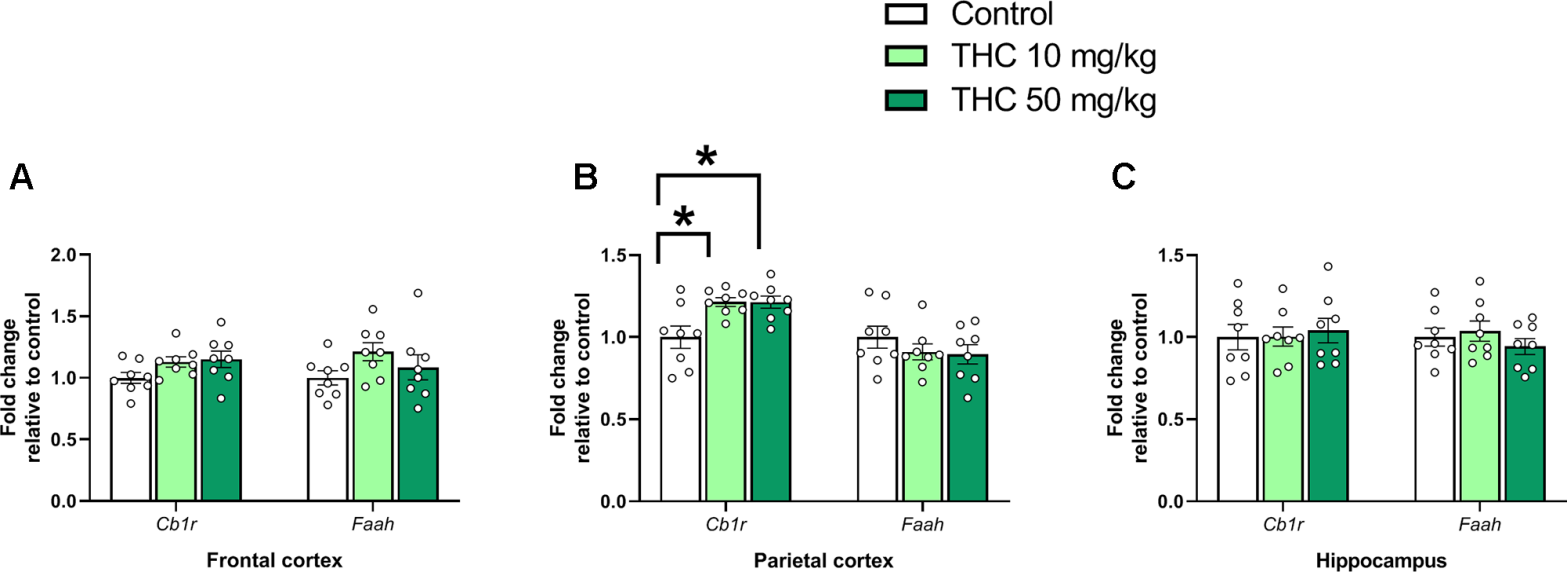
Endocannabinoid gene markers. Transcript levels of endocannabinoid genes *Cb1r* and *Faah* 24 h after PND 10 exposure to vehicle, 10 mg THC/kg or 50 mg THC/kg in the **(A)** frontal cortex, **(B)** parietal cortex, and **(C)** hippocampus. Statistical difference from *post hoc* test is indicated as * if p < 0.05 Height of bars represents the mean fold change ± SEM of 8 mice.

**Figure 3:**
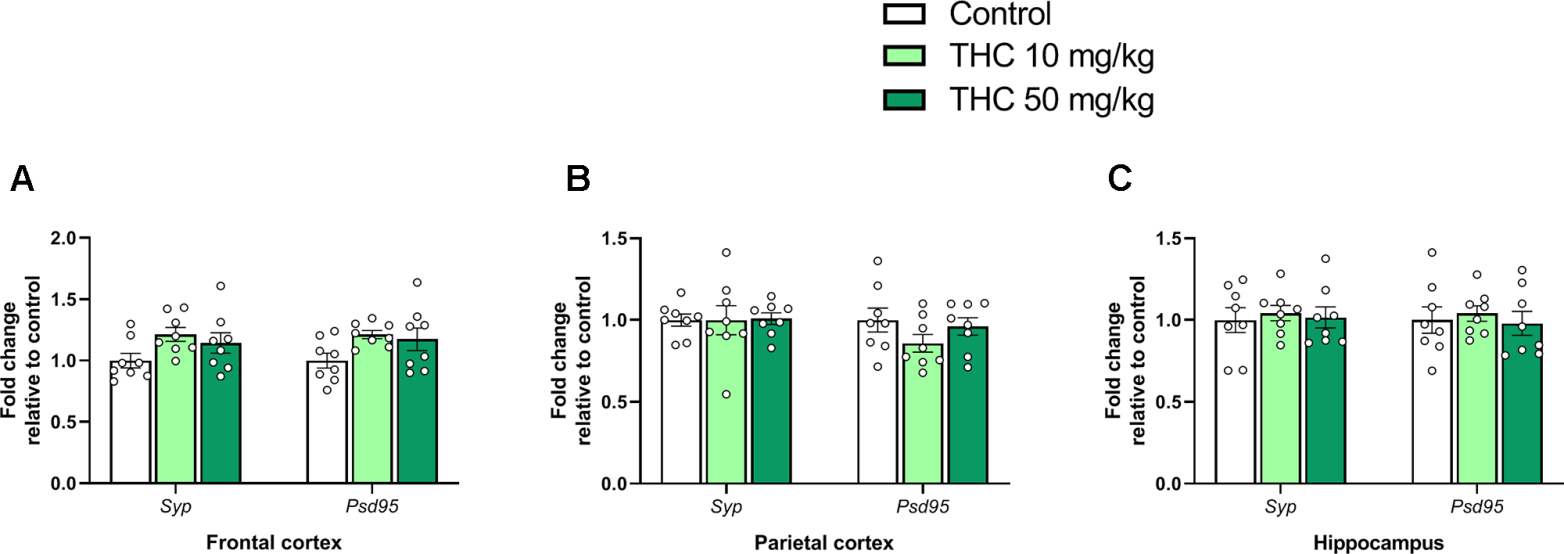
Synaptic density genes. Transcript levels of synaptic density gene markers *Syp* and *Psd95* 24 h after PND 10 exposure to vehicle, 10 mg THC/kg or 50 mg THC/kg in the **(A)** frontal cortex, **(B)** parietal cortex, and **(C)** hippocampus. Height of bars represents the mean fold change ± SEM of 8 mice.

There was an effect of THC on relative gene expression of *Cb1r* in the parietal cortex [F (2, 21) = 6.87, *p* = 0.0051, η_p_^2^ = 0.40] (**Figure 2B**). Tukey’s multiple comparison test revealed that mice neonatally exposed to both 10 mg/kg (p = 0.011) and 50 mg/kg (p = 0.011) had a significantly lower *Cb1r* relative transcription compared controls (**Figure 2B**). There was no significant effect on *Syp* or *Psd95* transcript levels in either the frontal cortex, the parietal cortex or in the hippocampus 24h after exposure (p > 0.05) (**Figure 3A–C**).

**Figure 4:**
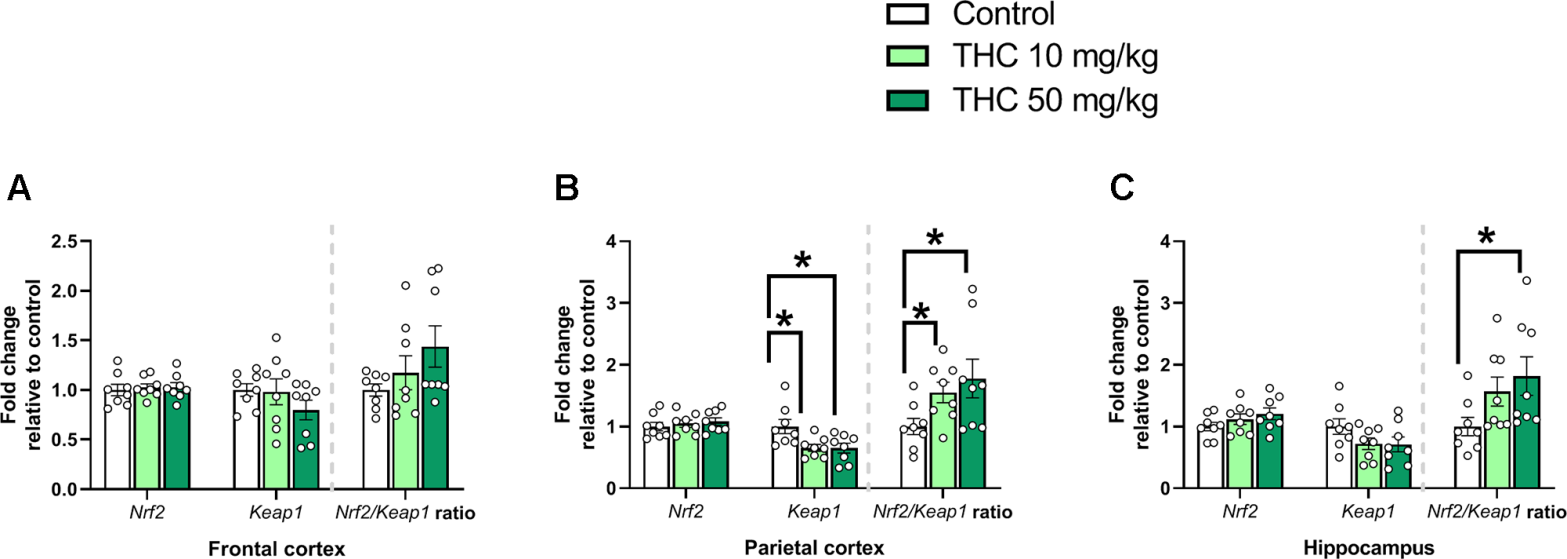
Oxidative stress genes. Transcript levels of oxidative stress genes *Nrf2* and *Keap1*, together with a *Nrf2/Keap1* expression ratio, 24 h after PND 10 exposure to vehicle, 10 mg THC/kg or 50 mg THC/kg in the **(A)** frontal cortex, **(B)** parietal cortex, and **(C)** hippocampus. Statistical difference from *post hoc* test is indicated as * if p < 0.05. Height of bars represents the mean fold change ± SEM of 8 mice.

There was an additional effect of THC treatment on the transcript levels of *Keap1* in the parietal cortex [F (2, 21) = 5.31, *p* = 0.014, η_p_^2^ = 0.34] (**Figure 4B**). Tukey’s multiple comparison test revealed that mice neonatally exposed to both 10 mg/kg (*p* = 0.029) and 50 mg/kg (*p* = 0.025) had a significantly lower relative transcription of *Keap1* compared to controls (**Figure 4B**). There was also an effect by treatment on the *Nrf2/Keap1* gene expression ratio in the parietal cortex [F (2, 21) = 5.31, *p* = 0.014, η_p_^2^ = 0.34] and hippocampus [F (2. 21) = 4.11, *p* = 0.031, η_p_^2^ = 0.28] (**Figures 4B, C**). In the parietal cortex both 10 mg THC/kg (*p* = 0.028) and 50 mg THC/kg (*p* = 0.025) increased the *Nrf2/Keap1* ratio 24 h after exposure (**Figure 4B**); in the hippocampus only 50 mg THC/kg (*p* = 0.033) increased the *Nrf2/Keap1* ratio 24 h after exposure (**Figure 4C**).

### ELISA

BAX protein expression is shown in **Table 2**. Student’s t-test revealed a significant increase of BAX in the frontal cortex 24 h after exposure to 10 mg THC/kg [t (12) = 2.7, *p* = 0.019, *d* = 0.9].

**Table 2:** Comparison of BAX levels in 3 different brain regions 24 h after exposure to either 10 mg THC/kg or vehicle on PND 10 in male mice.

Brain region	Controls (mean ± SEM)	THC 10 mg/kg (mean ± SEM)	p-value	Effect size (Cohen’s d)
*Frontal Cortex*	173.3 ± 22.04 (n = 7)	888.5 ± 362.5 (n = 7)	0.019*	0.9
*Parietal Cortex*	555.8 ± 274.9 (n = 6)	1144 ± 487.4 (n = 6)	0.74	0.9
*Hippocampus*	639.4 ± 310 (n = 7)	835.8± 334.1 (n = 7)	0.53	0.3

*Statistical difference from Student's t-test.

## Discussion

The present study demonstrates that a single dose of THC during postnatal brain development changed the transcript levels of genes involved in neurotrophic, endocannabinoid and oxidative stress signaling, and increased a marker of apoptosis, 24 h after exposure in male mice. Specifically, a single THC dose (50 mg/kg) into 10-day-old mice decreased the relative transcript levels of the neurotrophic receptor *Trkb* in both the frontal and parietal cortex and in the hippocampus. Effects on the *Nrf2-Keap1* axis were also found in both the parietal cortex (10 and 50 mg/kg) and hippocampus (50 mg/kg) following neonatal exposure to THC. Additionally, THC exposure (10 and 50 mg/kg) increased the transcript levels of *Cb1r* in the parietal cortex. An increase of pro apoptotic marker BAX was also detected in the frontal cortex 24 h after exposure.

It is well known that the endocannabinoid system is an emerging target of pharmacotherapy as the endocannabinoids are implicated in a manifold of both physiological and pathological processes (Di Marzo, 2018). The effects of THC is mediated *via* two receptor subtypes: CB_1_R and CB_2_R (Matsuda et al., 1990; Munro et al., 1993). The endogenous ligands of the endocannabinoid system are anandamide and 2-arachidonylglycerol (2-AG) (Devane et al., 1992; Mechoulam et al., 1995). The endocannabinoid system is already present during early brain development (Maccarrone et al., 2014) and is instrumental in many processes during this period (Berghuis et al., 2005; Berghuis et al., 2007; Harkany et al., 2007; Harkany et al., 2008; Mulder et al., 2008).

We have previously reported that a single dose of THC (both 10 and 50 mg/kg), injected into 10-day-old mice, affected adult spontaneous behavior and habituation rates in a new home environment (Philippot et al., 2016). This study demonstrated that *Trkb* gene expression was affected following 50 mg THC/kg in frontal cortex, parietal cortex, and hippocampus. TRKB, and its associated activator BDNF, are highly important in neuronal function as they are required for both neuronal survival and differentiation during brain development and in synaptic and behavioral plasticity in mature neurons, including hippocampal-dependent memory (Huang and Reichardt, 2003; Heldt et al., 2007; Barnes and Thomas, 2008). Interestingly, TRKB conditional knockout animals display impaired spatial learning (Minichiello et al., 1999). In line with the observed effects on *Trkb* transcript levels presented herein, there is already a known interaction between the endocannabinoid system and the BDNF–TRKB pathway — especially during brain development (Berghuis et al., 2005; D’Souza et al., 2009). If these transcriptional changes on neurotropic marker gene *Trkb*, following PND 10 exposure to THC, also could be detected on protein levels of TRKB remain unknown.

The transcriptional effect on *Trkb* following PND 10 exposure to THC is also interesting for the developmental neurotoxic evaluation of AAP; in a previous study, PND 10 exposure to AAP also decreased transcription of *Trkb* in mice (Philippot et al., 2018). Since both THC and AAP interact with the endocannabinoid system (Bertolini et al., 2006), together with the fact that PND 10 exposure to both these substances affect 1) adult spontaneous behavior and habituation rates and 2) decreased transcript levels of *Trkb* 24 *h* after exposure, there is a possibility that both these substances share important events in their respective AOP. This is in line with our hypothesis proposed elsewhere (Viberg et al., 2014; Philippot et al., 2017) where we suggested that PND 10 exposure to AAP (through its metabolite AM404) activate CB_1_R during brain development, which in turn, affects the BDNF–TRKB pathway. Additionally, the negative effect of PND 10 AAP exposure on adult spontaneous behavior and habituation rates was enhanced by simultaneous exposure to CB_1_R agonist WIN 55 212-2 (Philippot et al., 2018), further indicating the critical role of the endocannabinoid system during brain development.

An increase of the *Nrf2/Keap1* ratio was displayed in the hippocampus and in the parietal cortex 24 h after neonatal exposure to THC (50 mg/kg in hippocampus; 10 and 50 mg/kg in parietal cortex). The transcription factor NRF2 and its main negative regulator, KEAP1, are critical in the response to oxidative stress (Sies et al., 2017). The increase of the *Nrf2/Keap1* ratio therefore indicates a transcriptional up-regulation of cytoprotective elements as a response to cellular oxidative stressors such as reactive oxidative species (ROS). However, the effects of cannabinoids on oxidative stress are ambiguous. There are studies showing that cannabinoid exposures both have protective (Chen et al., 2005; Jia et al., 2014) and inducing (Wolff et al., 2015) effects on oxidative stress. There is a possibility that the increase in *Nrf2/Keap1* ratio was induced by a THC/CB_1_R-mediated alteration of the neurotrophic interaction between BDNF and TRKB. In line with this hypothesis it has been shown that the CB_1_R exert neuroprotective effects through *Bdnf* gene up-regulation (Khaspekov et al., 2004). However, whether the effects of neonatal exposure to THC on *Trkb* transcription are involved in the effects shown on oxidative stress markers are still unknown.

An increase in transcript levels of *Cb1r* in the parietal cortex was observed following PND 10 THC exposure, which may reflect an increase in the number of receptors. Even though a down-regulation of the receptor was expected following the exposure of an agonist, the up-regulation of *Cb1r* transcript levels may indicate the activation of another signaling system distinct from the endocannabinoid. For example, a lack of dopamine receptor D2 increased transcript levels of CB_1_R in mice (Thanos et al., 2011). However, short-term exposure to THC is expected to increase dopamine levels, not decrease; on the other hand, in situations where the brain is habituated to exogenous cannabinoid stimulation, a blunting effect on dopamine signaling can be expected (Bloomfield et al., 2016). We can only speculate that THC could blunt dopaminergic signaling (or another signaling system) and that this effect, in turn, up-regulates transcript levels of *Cb1r*. However, it seems less likely that a single dose of THC has an effect as such 24 h after exposure. In T-cells, an up-regulation of *Cb1r* transcript levels has been demonstrated following exposure to THC; in this study authors show that the up-regulation of *Cb1r* transcript levels are CB_2_R mediated (Borner et al., 2007). In another study using cultured striatal cells, an up-regulation of *Cb1r* transcripts was observed following exposure to different cannabinoids — an effect that was shown to be CB_1_R mediated (Laprairie et al., 2013). Similar effects on *Cb1r* transcription following exposure to cannabinoids has been observed in a mouse model of Huntington’s disease (a condition associated with an early down-regulation of the *Cb1r* gene)(Blázquez et al., 2010). Whether the up-regulation of *Cb1r* following PND 10 exposure depends on the timing of the exposure, an alternative signaling pathway distinct from the endocannabinoid system, CB_2_R activation or on which specific brain area that are investigated, remains to be investigated. In addition to the Cb1r transcript levels, we also investigated potential effect on the transcript levels of endocannabinoid-associated enzyme FAAH. However, no effect on *Faah* transcription was observed following PND 10 THC exposure. Nevertheless, the surprising increase of *Cb1r* transcript levels following THC exposure need to be further studied and could give important insights on the AOP of developmental exposure to THC.

Synaptic density was assessed in the frontal cortex, parietal cortex, and in the hippocampus, of the neonatal mouse by measuring transcript levels of two synaptic markers: SYP (presynaptic) and PSD-95 (postsynaptic). No effect on these markers was observed following treatments. In a previous study, on the other hand, we demonstrated that exposure to paracetamol on PND 10 affected transcript levels of *Syp* (Philippot et al., 2018). Nonetheless, it would be interesting if potential developmental effects on micromorphology and/or synaptic density following PND 10 exposures to both THC and AAP would be evaluated more thorough using e.g. (immuno)histological methods.

Higher levels of pro apoptotic marker BAX were found in the frontal cortex of mice exposed to 10 mg THC/kg on PND 10 compared to the controls 24 h after exposure. Pro apoptotic effector protein BAX is required for apoptosis (Wei et al., 2001). As a response to stress, BAX induces the release of cytochrome c and other pro-apoptotic factors from the mitochondria in turn leading to activation of caspases (Chipuk and Green, 2008). There is also a connection between BDNF–TRKB signaling and activation of BAX (Sun et al., 2012), thus the effects on BAX may be a result of the effect observed on the transcript levels of *Trkb* and/or *Nrf2/Keap1*.

As indicated by the effect size measurements, the effect of THC on all significantly changed transcript and protein levels, in general seem large (all was above η_p_^2^ > 0.14 or *d* > 0.8); it is nonetheless known that small sample sizes potentially inflate the estimated effect sizes. However, the estimated large effect sizes, together with significant *p*-values (*p* < 0.05), indicate that the effects on transcript levels presented herein neither are irrelevant, nor chance-derived.

The alterations in adult behavior observed following PND 10 THC exposure are in line with previously reviewed behavioral consequences (Campolongo et al., 2011; Higuera-Matas et al., 2015). However, common to these reviewed studies is that the cannabinoid exposure regimens are 1) lasting for several consecutive days or weeks and 2) that exposure occurs during gestation (or at least starting during gestation). As there are different critical periods during brain development, we argue that a single-day exposure to THC add toxicological data of high importance, not only to illustrate the potency of THC as a developmental neurotoxicant, but also to highlight the existence of short critical windows during brain development. In terms of translational toxicity between mice and humans, there are difficulties as mice are expected to have narrower, and perhaps more critical, windows of increased vulnerability during brain development compared to humans. In humans, key developmental processes are often prolonged over longer time spans compared to mice, thus weeks of exposure in mice may correspond to extreme exposure situations in a human setting. Therefore, the single-day exposure model used in this study may, in terms of translational relevancy between mouse and human, correspond to more realistic human exposures compared to rodent models where exposures lasting for several consecutive days/weeks.

In conclusion, we have shown that PND 10 exposure to a single dose of THC decreased transcript levels of neurotrophic receptor *Trkb*. We also conclude that PND 10 exposure to THC increased the *Nrf2/Keap1* ratio — a sign of oxidative stress — in both the parietal cortex and hippocampus. Increased transcript level of the *Cb1r* gene was also observed in the parietal cortex. We also detected increased levels of BAX protein in the frontal cortex. These results are important for many reasons: 1) we point out a critical window during brain development with increased sensitivity to THC exposure and begin to map out the potential mechanisms behind these effects, 2) there is a greater need for scientific understanding of both harms and benefits of THC usage as there are ongoing legalization/decriminalization initiatives regarding cannabis use, together with the fact that pharmaceutical companies are interested in using the endocannabinoid system as a target for pharmacotherapy (Pacher et al., 2006; Di Marzo, 2018), and finally 3) by comparing the effects of neonatal THC exposure to those of already observed following neonatal AAP exposure (which also affect the endocannabinoid system). We here suggest that both THC and AAP exposure on PND 10 may share key events in their respective AOP through endocannabinoid-mediated alterations of the BNDF-TRKB signaling pathway.

## Data Availability Statement

The datasets generated for this study are available on request to the corresponding author.

## Ethics Statement

The animal study was reviewed and approved by the Local ethical committee in Uppsala (UPPSALA djurförsöksetiska nämnd, Uppsala District Court, 75141, Uppsala).

## Author Contributions

GP, HV, and RF designed the experiments. GP and EF analyzed the data. GP wrote the manuscript. CT, GP, and EF performed most of the experiments. All authors critically reviewed and approved the final form of the manuscript.

## Conflict of Interest

The authors declare that the research was conducted in the absence of any commercial or financial relationships that could be construed as a potential conflict of interest.
